# A new data-driven mathematical model dissociates attractiveness from sexual dimorphism of human faces

**DOI:** 10.1038/s41598-020-73472-8

**Published:** 2020-10-06

**Authors:** Koyo Nakamura, Katsumi Watanabe

**Affiliations:** 1 Faculty of Science and Engineering, Waseda University, 3-4-1, Ohkubo, Shinjuku, Tokyo, 169-8555 Japan; 2 Japan Society for the Promotion of Science, Tokyo, Japan; 3 Keio Advanced Research Centers, Tokyo, Japan; 4 Art and Design, University of New South Wales, Sydney, Australia

**Keywords:** Psychology, Human behaviour

## Abstract

Human facial attractiveness is evaluated by using multiple cues. Among others, sexual dimorphism (i.e. masculinity for male faces/femininity for female faces) is an influential factor of perceived attractiveness. Since facial attractiveness is judged by incorporating sexually dimorphic traits as well as other cues, it is theoretically possible to dissociate sexual dimorphism from facial attractiveness. This study tested this by using a data-driven mathematical modelling approach. We first analysed the correlation between perceived masculinity/femininity and attractiveness ratings for 400 computer-generated male and female faces (Experiment 1) and found positive correlations between perceived femininity and attractiveness for both male and female faces. Using these results, we manipulated a set of faces along the attractiveness dimension while controlling for sexual dimorphism by orthogonalisation with data-driven mathematical models (Experiment 2). Our results revealed that perceived attractiveness and sexual dimorphism are dissociable, suggesting that there are as yet unidentified facial cues other than sexual dimorphism that contribute to facial attractiveness. Future studies can investigate the true preference of sexual dimorphism or the genuine effects of attractiveness by using well-controlled facial stimuli like those that this study generated. The findings will be of benefit to the further understanding of what makes a face attractive.

## Introduction

Attractiveness is one of the most influential human facial attributes^1,2^. The facial features that drive perceived attractiveness have been extensively studied in the field of social and cognitive psychology^3,4^. To date, several facial morphologies and skin properties have been identified as critical cues to attractiveness, including averageness, symmetry, and skin colour^5,6^. From the perspective of evolutionary psychology, humans have become sensitive to the facial features signalling health and sexual maturity for reproductive success^7–9^. There is some evidence that facial averageness and symmetry signal high genetic quality^10,11^, thus leading to a preference for average-looking or symmetric faces.

Sexual dimorphism (i.e. masculinity for male faces/femininity for female faces) also affects perceived attractiveness^12–14^. Male and female faces are differentiated at puberty under the influence of sexual hormones such as oestrogen and androgen. Although male faces look more masculine and female faces look more feminine, it also differs within the sexes. The higher sex-typicality of sexual dimorphism is proposed to be linked to actual health^7,9^. For example, men with masculine facial features and women with feminine facial features are likely to be healthier than men with feminine faces and women with masculine faces^9^. Accordingly, male faces with more masculine traits^13,15^ and female faces with more feminine traits^12,16^ are perceived as more attractive. In particular, the preference for femininity in female faces is widespread across cultures and ages^12,16,17^. In contrast, the preference for masculinity in male faces varies more; some studies demonstrated a masculinity preference^13,15,18,19^ while some other studies showed a femininity preference^12,16^, or no obvious relationship^20^. Evolutionary psychologists have posited that the sex-typical facial traits might be preferred because they signal a high genetic quality in terms of potential mating and health (i.e. good gene traits^3,11^). However, masculinity in male faces can be associated with negative personality traits and behaviours; male-looking facial features are generally less preferred because these features conceive emotionally negative male personalities such as aggressiveness or dominance^12,21^. Likewise, highly masculine faces are perceived as less emotionally warm and less cooperative than more feminine faces^12^. Some studies showed that highly masculine men are more likely to have marital problems and divorce^22^, and are less likely to feel sympathetic to infants^23^. In other words, feminine-looking men, more than masculine-looking men, are willing to devote their parental investment to their offspring in order to increase the chance of their offspring’s survival (i.e. good dad traits;^24^). Such a trade-off between preference for a good gene and a good dad makes male facial attractiveness judgments more complicated.

For decades, averageness, symmetry, and sexual dimorphism have been well-documented as the major cues to attractiveness^6,17^. However, there are potential methodological limitations to previous facial attractiveness studies. For example, many studies on the effect of sexual dimorphism on perceived attractiveness have employed face morphing methods to increase or decrease facial femininity/masculinity. Such methods eliminate natural variations in other important cues^25^, which are often important determinants of facial impressions^1,26^. Recent data-driven computational modelling studies proposed that facial attractiveness is not solely determined by the combination of the abovementioned attractiveness cues^14,26^. Data-driven methods are used to build a model to represent how facial features in a multidimensional face space (e.g. shape and skin properties) vary along perceived attractiveness with no a priori hypotheses or constraints^1,27^. Recently, Nakamura and Watanabe^26^ built a data-driven mathematical model of East-Asian facial attractiveness by sampling a number of computer-generated faces that differ in multiple facial feature dimensions and found that the facial features of attractive faces include feminine traits such as larger eyes, smaller noses, and brighter skin, irrespective of the sex of faces. The results are consistent with previous evidence that femininity is preferred when judging both male and female facial attractiveness^12,16,21^. Furthermore, Said and Todorov^14^ compared the prediction performance of the data-driven computational model and the hypothesis-driven model, incorporating averageness and sexual dimorphism. The results showed a higher prediction performance of the data-driven model over the hypothesis-driven model^14^. This suggests that data-driven modelling has great potential for discovering as yet unidentified facial cues to attractiveness, which have thus far been overlooked by hypothesis-driven research.

As reviewed above, sexual dimorphism has a great impact on facial attractiveness judgments^12,13,16^. Nevertheless, perceived attractiveness is not necessarily determined solely by the levels of sexual dimorphism. Given that a data-driven mathematical model predicts perceived attractiveness better than the combination of the well-documented attractiveness cues^14^, it is theoretically possible to identify facial cues to attractiveness with controlling for sexual dimorphism. Dissociating attractiveness from sexual dimorphism helps us to reveal and visualise as yet unidentified facial cues to attractiveness other than sexual dimorphism. Cornwell et al.^28^ suggested that facial attractiveness can be manipulated independently of sexual dimorphism by generating face prototypes with different attractiveness and similar masculinity/femininity. However, this manipulation was successful only for male faces but not for female faces^28^, in part because the orthogonalization of ratings of attractiveness and sexual dimorphism was incomplete. In this study, extending the data-driven attractiveness model built by the authors^26^, we aimed to identify an attractiveness dimension orthogonal to sexual dimorphism for both male and female faces. We first confirmed that there was a significant correlation between perceived attractiveness and sexual dimorphism (masculinity/femininity) even for computer-generated East-Asian faces, as demonstrated in the previous studies^12,16^. Then we extracted a sexual dimorphism dimension in the same way that Nakamura and Watanabe^26^ built an attractiveness dimension. Dissociating attractiveness from sexual dimorphism enabled us to generate faces of varying attractiveness without changing sexual dimorphism and faces with varying sexual dimorphism without changing attractiveness.

The rest of the paper is organised as follows: The method of the first experiment outlined above is explained, followed by its results and a discussion thereof. Then, the method of the second experiment outlined above is explained, followed by its results and a discussion thereof. We conclude with a general discussion of the whole study.

## Experiment 1

### Methods

#### Participants

Ten Japanese male and 10 Japanese female participants were recruited for a model building of a sexual dimorphism dimension (mean age = 21.45, *SD* = 1.47). For a model validation test, a separate group of eight Japanese male and eight Japanese female participants were recruited (mean age = 21.45, *SD* = 2.69). All participants had normal or corrected-to-normal vision and were naïve to the purpose of the study. The study was approved by the institutional review board of Waseda University (2015-033). All procedures were carried out in accordance with the Declaration of Helsinki. Written informed consent was obtained from all participants in advance. The sample sizes were identical to that of Nakamura and Watanabe^26^ in order to perform data-driven calculations based on the same sample sizes.

#### Apparatus and stimuli

We used 200 male and 200 female computer-generated East-Asian faces that were originally generated by Nakamura and Watanabe^26^ with FaceGen Modeller (Singular Inversions, Toronto, Canada). All the faces were viewed front-on and were emotionally neutral. The 400 individual faces were represented by 100 orthogonal feature dimensions (50 shape dimensions and 50 reflectance dimensions; for detail see Nakamura and Watanabe^26^).

#### Procedure

Model building. To build a data-driven mathematical model of sexual dimorphism, the 20 participants were asked to rate sexual dimorphism (i.e., masculinity/femininity) of 400 East-Asian computer-generated faces (200 female faces). In the rating task, following a fixation cross for 500 ms, a face appeared in the centre of the screen. The participants were asked to rate the perceived masculinity/femininity on a scale ranging from 1 (extremely feminine) to 9 (extremely masculine) without time constraint. The rating was made based on subjective but relative criteria. The participants completed two separate blocks, within each of which the sex of faces was fixed. Within a session, the faces were presented in a random order, and the order of the blocks was counter-balanced across participants. We defined sexual dimorphism based on subjective rating scores for masculinity/femininity, instead of the gender control value of FaceGen Modeller. This was because (i) defining sexual dimorphism as subjective rating scores would make treating attractiveness and sexual dimorphism in the same way and (ii) not only sexual dimorphic physical features but also other physical features affect masculinity/femininity judgements^29^.

Model validation. In order to build a data-driven model representing how facial shape and reflectance vary with perceived masculinity/femininity, we applied Nakamura and Watanabe’s^26^ data-driven calculations to the rating scores of sexual dimorphism (for methodological details, see Nakamura and Watanabe^26^). In the study, the 400 computer-generated faces were represented in 100-dimeensional face space (50 shape and 50 reflectance dimensions). Nakamura and Watanabe^26^ modelled the attractiveness dimension as linear combinations of the 100 face vectors, that is, the best linear fit of the attractiveness rating scores collected from the observers. Their results showed that the model-based manipulation of facial shape and reflectance allowed for almost linear changes in perceived attractiveness for both male and female faces.

**Figure 1:**
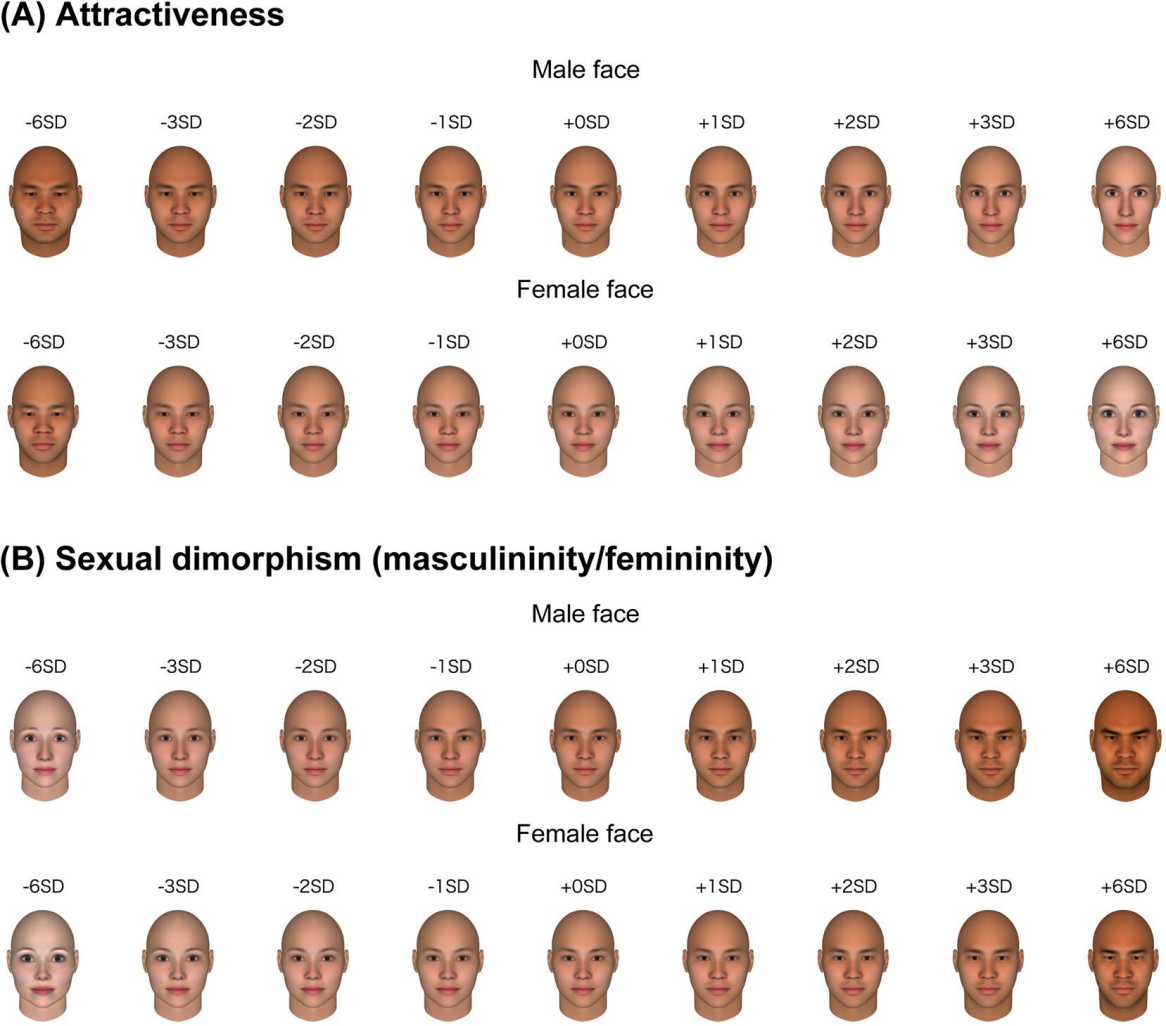
Data-driven mathematical model of facial attractiveness (**A**) and sexual dimorphism (**B**). The extent of the facial manipulation is presented in SD units. For visual illustration, + /− 6 SD faces are also presented, although these faces have not been used in Experiment 1. The face images were generated with FaceGen Modeller (https://facegen.com/).

Because the interrater reliability (indexed by Cronbach’s alpha) was high for both male (α = 0.98) and female (α = 0.98) faces, we computed mean masculinity/femininity rating scores across all the participants and the mean rating scores were standardised. For the attractiveness rating scores reported in Nakamura and Watanabe^26^, the interrater reliability was high for both male (α = 0.93) and female faces (α = 0.95). For illustration, the original attractiveness model built in Nakamura and Watanabe^26^ and a sexual dimorphism model are presented in Fig. 1.

To validate our model of the sexual dimorphism dimension, we applied the sexual dimorphism manipulation to 20 novel randomly generated faces, which were used in the model validation study in Nakamura and Watanabe^26^. With this manipulation, we created seven versions of the 20 faces, varying the sexual dimorphism level from − 3 (feminine) to + 3 (masculine) in an *SD* unit. The 16 participants were asked to rate perceived masculinity/femininity on a 9-point Likert scale (1: extremely feminine, 9: extremely masculine). The rating was made based on subjective but relative criteria. The participants completed two separate blocks, within each of which the sex of faces was fixed. Within a session, the faces were presented in a random order, and the order of the blocks was counter-balanced across participants.

#### Data analysis

The rating scores of sexual dimorphism were analysed with hierarchical Bayesian regression models implemented using the ‘*rstan*’ (version 2.19.3)^30^ and ‘*brms*’ (version 2.12.0)^31^ packages in R (version 3.5.1)^32^. In the regression model, we included face exaggeration (SD) as a continuous fixed effect because our main interest was to test the effect of face exaggeration on the rating scores. To examine the modulating factors of face exaggeration, we also included the sex of faces and the sex of raters as fixed effects (i.e., the interaction effect involving face exaggeration). We also entered a quadratic face exaggeration term as we anticipated the relationship of the rating scores and face exaggeration to be in part curvilinear. In addition, we maximised the random effect structure justified by the data structure, and thus random by-participant intercepts and slopes and by-face intercepts and slopes for all the fixed effects and its interactions were all included. We determined a best-fitted model by calculating the WAIC (widely applicable information criterion) of all possible combinations between models with the face exaggeration and the modulating factors.

Parameter estimation was performed with 13,000 iterations, 3000 burn-in samples, and 4 chains. The value of Rhat for all parameters almost equalled 1.0, indicating convergence across the 4 chains. The expected a posteriori (EAP) and 95% credible interval (CrI) were used to compute representative values for the estimated parameters.

### Results and discussion

#### Validation of sexual dimorphism dimension

**Figure 2:**
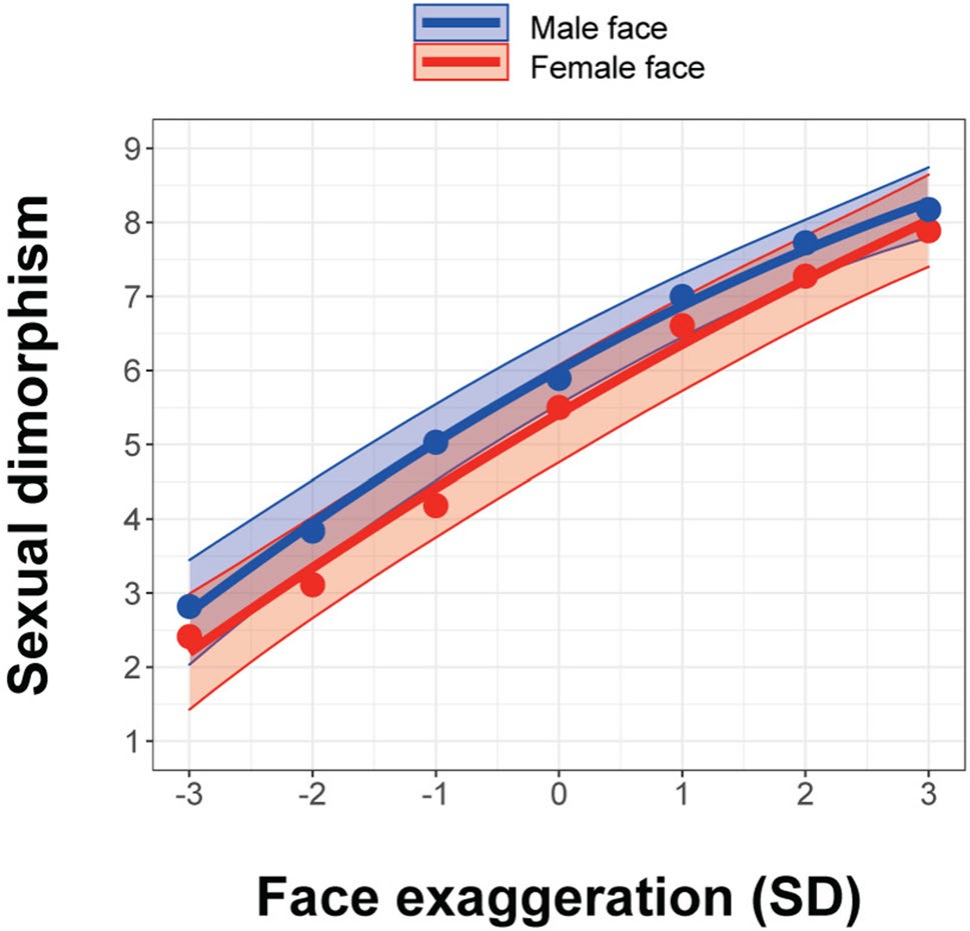
Validation of the data-driven mathematical model for the sexual dimorphism dimension. Plotted data points are mean rating scores across participants. Sexual dimorphism was rated on a scale ranging from 1 (extremely feminine) to 9 (extremely masculine). Lines and error bars indicate the predicted values and its 95% CrI from the best-fitted hierarchical regression model.

In order to confirm that data-driven manipulation of facial features associated with sexual dimorphism would successfully predict perceived masculinity/femininity, we analysed the rating scores of sexual dimorphism with the hierarchical Bayesian regression models. As a result of model selection as indexed by the WAIC, the model that incorporated face exaggeration (SD) as both linear and quadratic terms, the sex of faces, and its interaction was selected as a best-fitted model (WAIC = 6864.94; see also Table S1). The perceived masculinity/femininity rating score for male faces significantly changed with the model-based facial feature manipulation irrespective of the sex of raters (Linear term = 0.92, 95% CrI = [0.78 to 1.06]; Quadratic term =  −0.06, 95% CrI = [− 0.10 to − 0.02]; Intercept = 6.01, 95% CrI = [5.54 to 6.48], Fig. 2). The perceived masculinity/femininity rating score for female faces also significantly changed with the model-based facial feature manipulation irrespective of the sex of raters (Linear term = 0.97, 95% CrI = [0.85 to 1.09]; Quadratic term =  −0.03, 95% CrI = [− 0.08 to 0.01]). The sex of faces had no significant effect on the rating (β =  −0.59, 95% CrI = [− 1.24 to 0.06]) and the effect of face exaggeration did not significantly differ between male and female faces (Linear term = 0.05, 95% CrI = [− 0.08 to 0.17]; Quadratic term = 0.02, 95% CrI = [− 0.03 to 0.07]). For the inter-rater reliability of the ratings, the intraclass correlation coefficient (ICC2) was 0.69 (*F*(139, 2085) = 45.78, *p* < 0.001, 95% CI = [0.64 to 0.74]), indicating the ratings were highly reliable across the participants. Thus, we confirmed that the data-driven mathematical model of sexual dimorphism for male and female faces was successfully built.

#### Correlation between perceived masculinity/femininity and attractiveness

**Figure 3:**
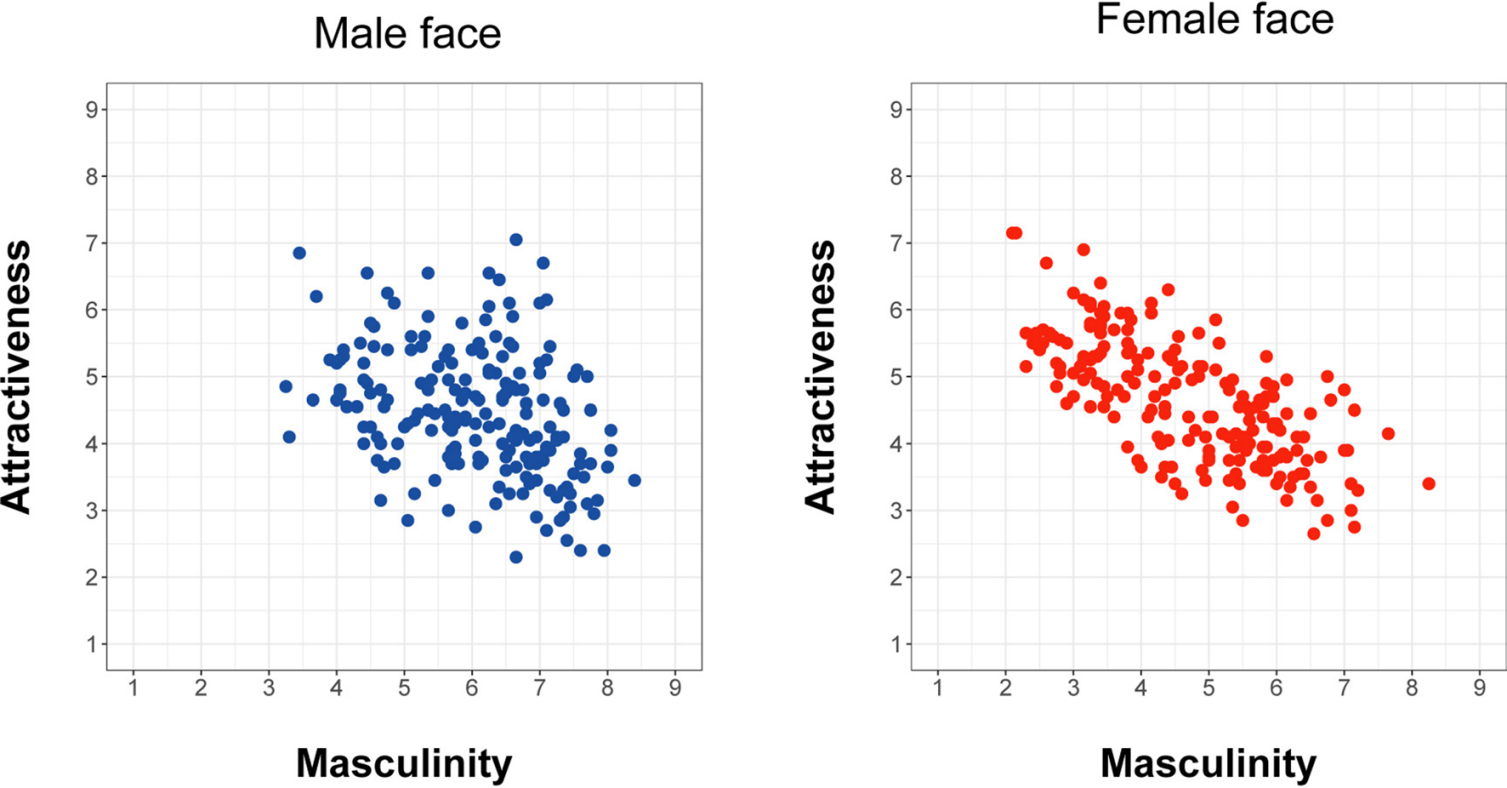
Scatter plots of the ratings of attractiveness and sexual dimorphism. The attractiveness ratings were measured in Nakamura and Watanabe (2019), while the sexual dimorphism ratings were measured in Experiment 1.

We calculated the correlation coefficients between the ratings of sexual dimorphism in the experiment and attractiveness measured in Nakamura and Watanabe^26^. The correlation coefficients were calculated separately for male and female faces, though the rating scores given by male and female participants were aggregated because of high inter-rater agreements. The results showed that perceived masculinity is negatively correlated with attractiveness (Fig. 3) for both male faces (ρ =  −0.37, 95% CrI = [− 0.49 to −0.25]) and female faces (ρ =  −0.70, 95% CrI = [− 0.76 to − 0.62]). In addition, the negative correlation between perceived masculinity and attractiveness was significantly higher for male faces than female ones (Δρ =  −0.32, 95% CrI = [− 0.46 to −0.19]). The results are consistent with the previous findings^12,16^; thus we replicated the preference for facial femininity by using computer-generated East-Asian faces. A relatively weak correlation between attractiveness and masculinity for male faces is reasonable, given that there is a trade-off between preference for good gene (masculinity) and good dad (femininity)^24^.

**Figure 4:**
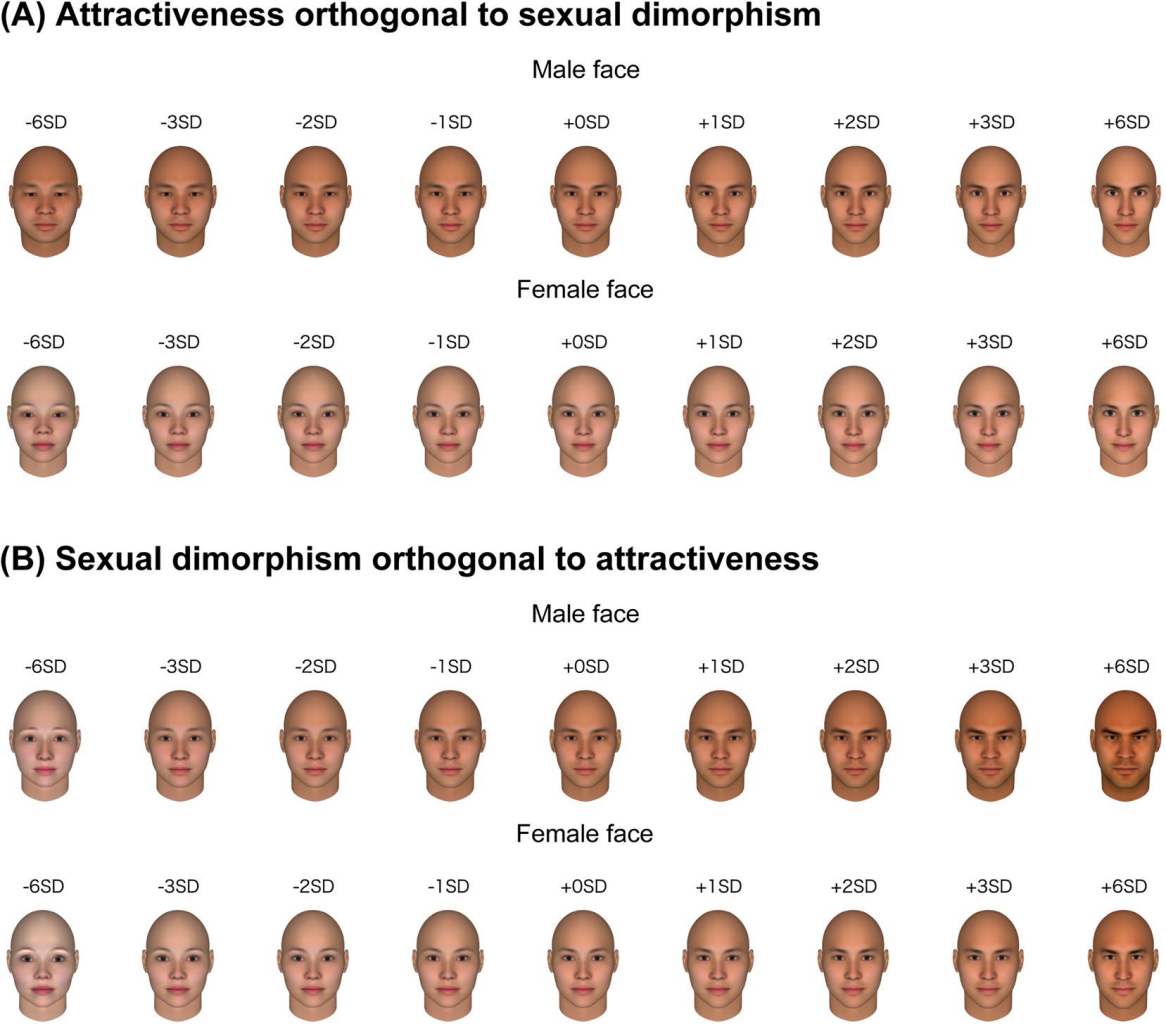
Data-driven computational model of attractiveness orthogonal to sexual dimorphism (**A**) and sexual dimorphism orthogonal to attractiveness (**B**). The extent of the facial manipulation is presented in SD units. For visual illustration, + /− 6 SD faces are also presented, although these faces have not been used in Experiment 2. The face images were generated with FaceGen Modeller (https://facegen.com/).

Combining the sexual dimorphism dimension built in the present experiment with the attractiveness dimension built in Nakamura and Watanabe^26^, facial features can be exaggerated along both dimensions or along a single dimension with the other dimensions remaining unchanged. More precisely, we achieved the latter manipulation by the orthogonalisation of the attractiveness vectors to the sexual dimorphism vectors, and vice versa^1,33^. For illustration, we presented nine versions of average male and female faces that were transformed either along (i) the attractiveness dimension controlling for sexual dimorphism or (ii) the sexual dimorphism dimension controlling for attractiveness with five exaggeration levels in Fig. 4. In Experiment 2, we presented a set of novel male and female faces that differed in the above-mentioned dimensions in order to confirm that attractiveness and sexual dimorphism can be dissociable.

## Experiment 2

### Methods

#### Participants

Thirty-two Japanese male and 32 Japanese female participants were recruited (mean age = 20.70, *SD* = 2.29). None of the participants took part in Experiment 1 in this study nor the experiment of Nakamura and Watanabe^26^. The study was approved by the institutional review board of Waseda University (2015-033). All procedures were carried out in accordance with the Declaration of Helsinki. Written informed consent was obtained from all participants in advance. In Experiment 2, participants were randomly allocated to one of four conditions as explained in the following section. The sample size was set to have 16 participants for each condition, which is the same as that of Experiment 1 (Model validation). All other conditions were the same as in Experiment 1. One male participant who performed the attractiveness rating task of the faces varying the attractiveness orthogonal to sexual dimorphism dimension was excluded from the data analysis. This was because his average response times was 594 ms whereas the other participants made the rating for 2343 ms on average, and we did not take his ratings reliable.

#### Apparatus and stimuli

In Experiment 2, we used 20 novel faces (10 female faces), which were used in neither the model building of the attractiveness dimension^26^ nor sexual dimorphism (Experiment 1). The faces were transformed along either (i) attractiveness dimension orthogonal to sexual dimorphism, and (ii) sexual dimorphism orthogonal to attractiveness. For the attractiveness dimension orthogonal to sexual dimorphism, we calculated the attractiveness vectors orthogonal to the sexual dimorphism vectors in a similar manner as Oh et al.^1^ and Buck and Todorov^33^ did, and the faces were exaggerated in seven levels in an SD unit (− 3, − 2, − 1, 0, + 1, + 2, + 3 SD), as was done in Nakamura and Watanabe^26^. In a similar vein, the faces were exaggerated in the sexual dimorphism orthogonal to the attractiveness dimension in seven levels. Applying the two types of transformation with seven levels to the 20 novel faces, we generated 280 faces in total. The experimental settings were identical to those used in Experiment 1.

#### Procedure

Model validation. Half of the participants were randomly allocated to the attractiveness rating task, and the other half were allocated to the sexual dimorphism rating task. In both rating tasks, the same number of male and female participants were allocated. On each rating task, the participants were presented with either of two types of face transformation.

In the attractiveness rating task, the participants rated the facial attractiveness of 70 male and 70 female faces on a 9 point-Likert scale (1: least attractive, 9: most attractive). In the sexual dimorphism rating task, the participants rated the perceived masculinity/femininity of 70 male and 70 female faces on a 9 point-Likert scale (1: extremely feminine, 9: extremely masculine). On each task, the participants completed two separate blocks, within each of which the sex of faces was fixed. The other parts of procedure were identical to those of Experiment 1.

#### Data analysis

The rating scores of attractiveness and sexual dimorphism were analysed separately with hierarchical Bayesian regression models as was done in Experiment 1. To simplify the model, we performed the regression analyses separately for the two types of face transformation. All parameter estimations and model selection processes were identical to those used in Experiment 1.

### Results and discussion

#### Attractiveness orthogonal to sexual dimorphism

**Figure 5:**
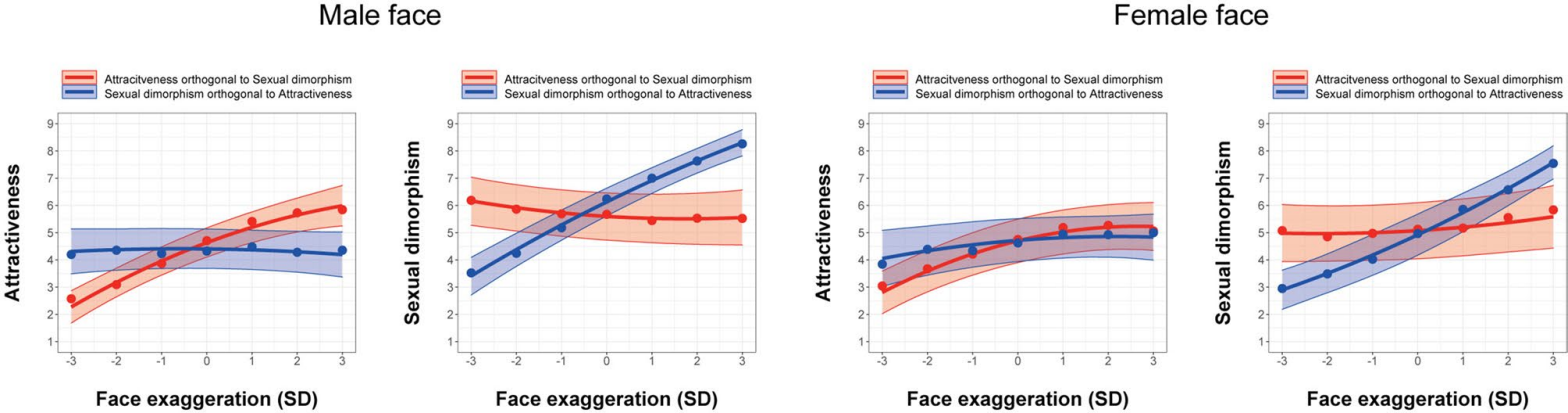
Validation of the data-driven mathematical model for the attractiveness orthogonal to sexual dimorphism and the sexual dimorphism orthogonal to attractiveness dimensions (left two panels for male faces, right two panel for female faces). Plotted data points are mean rating scores across participants. Lines and error bars indicate the predicted values and its 95% CrI from the best-fitted hierarchical regression model.

For the attractiveness rating scores with the faces exaggerated along the attractive dimension, the model that incorporated face exaggeration (SD) as both linear and quadratic terms, the sex of faces, and its interaction was selected as a best-fitted model as a result of the model selection (WAIC = 7101.69; see also Table S1). The attractiveness rating score for male faces significantly changed with the model-based facial feature manipulation irrespective of the sex of raters (Linear term = 0.62, 95% CrI = [0.48 to 0.75]; Quadratic term =  −0.06, 95% CrI = [− 0.09 to − 0.02]; Intercept = 4.63, 95% CrI = [4.10 to 5.17], Fig. 5). The attractiveness rating score for female faces also significantly changed with the model-based facial feature manipulation irrespective of the sex of raters (Linear term = 0.40, 95% CrI = [0.31 to 0.50]; Quadratic term =  −0.08, 95% CrI = [− 0.11 to − 0.04]). Although the sex of faces had no significant effect on the rating (β = 0.07, 95% CrI = [− 0.78 to 0.90]), the effect of linear face exaggeration was larger for male faces than female faces (Linear term =  −0.22, 95% CrI = [− 0.37 to − 0.07]; Quadratic term =  −0.02, 95% CrI = [− 0.07 to 0.02]). For the inter-rater reliability of the ratings, the intraclass correlation coefficient (ICC2) was 0.41 (*F*(139, 1946) = 13.43, *p* < 0.001, 95% CI = [0.36 to 0.47]), indicating the rating was moderately reliable across the participants.

For the sexual dimorphism rating scores, the model that incorporated face exaggeration (SD) as both linear and quadratic terms, the sex of faces, and its interaction was selected as a best-fitted model as a result of the model selection (WAIC = 7919.60; see also Table S1), although the WAIC of the model without the quadratic face exaggeration term was almost equivalent to that of the best-fitted model (WAIC = 7919.86). The sexual dimorphism rating score for male faces did not significantly change with the model-based facial feature manipulation irrespective of the sex of raters (Linear term =  −0.10, 95% CrI = [− 0.24 to 0.04]; Quadratic term = 0.03, 95% CrI = [0.00 to 0.06]; Intercept = 5.60, 95% CrI = [4.73 to 6.46], Fig. 5). The sexual dimorphism rating score for female faces also did not significantly change with the model-based facial feature manipulation irrespective of the sex of raters (Linear term = 0.10, 95% CrI = [− 0.01 to 0.21]; Quadratic term = 0.02, 95% CrI = [− 0.01 to 0.05]). Although the sex of faces had no significant effect on the rating (β =  −0.52, 95% CrI = [−1.88 to 0.83]), the effect of linear face exaggeration was larger for female faces than male faces (Linear term = 0.20, 95% CrI = [0.05 to 0.35]; Quadratic term =  −0.01, 95% CrI = [− 0.05 to 0.04]), reflecting a slight tendency of being female attractive faces perceived as more masculine. For the inter-rater reliability of the ratings, the intraclass correlation coefficient (ICC2) was 0.38 (*F*(139, 2085) = 11.02, *p* < 0.001, 95% CI = [0.33 to 0.43]), indicating the rating was moderately reliable across the participants.

Taken these results together, we found that even after controlling for the facial features associated with sexual dimorphism, attractiveness can be modulated by the data-driven face transformation while perceived masculinity/femininity remains unchanged. The data-driven attractiveness manipulation was successful for both male and female faces although the linear change in perceived attractiveness was larger for male faces than for female faces.

#### Sexual dimorphism orthogonal to attractiveness

For the attractiveness rating scores with the faces exaggerated along the sexual dimorphism dimension, the model that incorporated face exaggeration (SD) as both linear and quadratic terms, the sex of faces, its interaction, and the sex of raters was selected as a best-fitted model as a result of the model selection (WAIC = 8402.69; see also Table S1), although the WAIC of the model without the quadratic face exaggeration term was almost equivalent to that of the best-fitted model (WAIC = 8402.94). The attractiveness rating score for male faces did not significantly change with the model-based facial feature manipulation irrespective of the sex of raters (Linear term =  −0.02, 95% CrI = [− 0.16 to 0.12]; Quadratic term =  −0.02, 95% CrI = [− 0.05 to 0.01]; Intercept = 4.40, 95% CrI = [3.69 to 5.14], Fig. 5). The attractiveness rating score for female faces also did not significantly change with the model-based facial feature manipulation irrespective of the sex of raters (Linear term = 0.13, 95% CrI = [0.00 to 0.26]; Quadratic term =  −0.03, 95% CrI = [− 0.06 to 0.00]). The sex of faces had no significant effect on the rating (β = 0.31, 95% CrI = [− 0.55 to 1.18]), and the effect of face exaggeration did not significantly differ between male and female faces (Linear term =  − 0.15, 95% CrI = [− 0.05 to 0.35]; Quadratic term =  − 0.01, 95% CrI = [−0.06 to 0.04]). For the inter-rater reliability of the ratings, the intraclass correlation coefficient (ICC2) was 0.14 (*F*(139, 2085) = 4.24, *p* < 0.001, 95% CI = [0.11 to 0.18]), indicating the rating was less reliable across the participants.

For the sexual dimorphism rating scores, the model that incorporated face exaggeration as both linear and quadratic terms, the sex of faces, the sex of raters, and its interaction was selected as a best-fitted model (WAIC = 7391.79, see also Table S1). The sexual dimorphism rating score for male faces significantly changed with the model-based facial feature manipulation irrespective of the sex of raters in a linear fashion (Linear term = 0.81, 95% CrI = [0.70 to 0.93]; Quadratic term =  −0.03, 95% CrI = [− 0.06 to 0.00]; Intercept = 6.13, 95% CrI = [5.61 to 6.64], Fig. 5). The sexual dimorphism rating score for female faces also significantly changed with the model-based facial feature manipulation irrespective of the sex of raters (Linear term = 0.78, 95% CrI = [0.68 to 0.88]; Quadratic term = 0.04, 95% CrI = [−0.01 to 0.08]). Moreover, male faces were rated as being more masculine than female faces (β =  −1.21, 95% CrI = [− 2.13 to − 0.27]), and the effect of quadratic face exaggeration was larger for female faces than male faces (Linear term =  −0.04, 95% CrI = [−0.19 to 0.12]; Quadratic term = 0.07, 95% CrI = [0.01 to 0.12]). For the inter-rater reliability of the ratings, the intraclass correlation coefficient (ICC2) was 0.64 (*F*(139, 2085) = 30.40, *p* < 0.001, 95% CI = [0.59 to 0.69]), indicating the rating was highly reliable across the participants.

Taken these results together, we found that even after controlling for the facial features associated with attractiveness, perceived masculinity/femininity can be modulated by the data-driven face transformation while perceived attractiveness remains unchanged.

#### Comparison of the effect of face exaggeration on perceived attractiveness and sexual dimorphism

To compare the effect of linear and quadratic face exaggeration on perceived attractiveness and sexual dimorphism, we sampled the coefficients of linear and quadratic face exaggeration from the posterior distributions from each regression model. We then calculated the generated quantities by subtracting the coefficient of face exaggeration in the sexual dimorphism orthogonal to attractiveness model from that in the attractiveness orthogonal to sexual dimorphism model, separately for male and female faces. The generated quantities are summarised in Table 1.

**Table 1:** Generated quantities of the coefficients of face exaggeration.

Rating	Sex of faces	Hypothesis	Linear term EAP [95% CrI]	Quadratic term EAP [95% CrI]
Attractiveness	Male faces	β_attractiveness orthogonal to sexual dimorphism_ − β_sexual dimorphism orthogonal to attractiveness_	0.64 [0.44 to 0.83]	− 0.03 [− 0.08 to 0.01]
Attractiveness	Female faces	β_attractiveness orthogonal to sexual dimorphism_ − β_sexual dimorphism orthogonal to attractiveness_	0.27 [0.11 to 0.43]	− 0.04 [− 0.09 to 0.00]
Sexual dimorphism	Male faces	β_sexual dimorphism orthogonal to attractiveness_ − β_attractiveness orthogonal to sexual dimorphism_	0.92 [0.74 to 1.09]	− 0.06 [− 0.10 to − 0.02]
Sexual dimorphism	Female faces	β_sexual dimorphism orthogonal to attractiveness_ − β_attractiveness orthogonal to sexual dimorphism_	0.68 [0.53 to 0.82]	0.01 [− 0.03 to 0.06]

For the attractiveness rating scores, the coefficient of linear face exaggeration in the attractiveness orthogonal to sexual dimorphism model was significantly larger than that in the sexual dimorphism orthogonal to attractiveness model for both male and female faces. No such significant differences were seen for the coefficient of quadratic face exaggeration. For the sexual dimorphism rating scores, the coefficient of face exaggeration in the sexual dimorphism orthogonal to attractiveness model was significantly larger than that in the attractiveness orthogonal to sexual dimorphism model for both male and female faces. The coefficient of quadratic face exaggeration was significantly smaller than that in the attractiveness orthogonal to sexual dimorphism model for male faces, indicating sexual dimorphism rating in the sexual dimorphism orthogonal to attractiveness model was changed in an inverted U-shaped fashion more than that in the attractiveness orthogonal to sexual dimorphism rating. No such significant difference was seen for female faces (Table 1). These results indicate that the face transformation along one dimension orthogonal the other dimension provided larger changes in the rating scores on one than the other. It is of note that the changes in the rating scores were larger for male faces than female faces. This might be due to the higher correlation between attractiveness and sexual dimorphism rating scores for female faces (ρ =  −0.70) than male faces (ρ =  −0.37). It is likely that the facial features driving both attractiveness and sexual dimorphism were left out by face vector orthogonalisation more largely for female faces than male faces, thereby providing smaller changes in the ratings for female faces^15^.

## General discussion

This study aimed to test whether there are facial cues to attractiveness that are dissociable from sexual dimorphism by using data-driven mathematical modelling. Studies have shown that perceived femininity and masculinity affect attractiveness^3,4^. In particular, facial femininity is preferred as attractive features more often than facial masculinity^12,16^, possibly because masculinity is associated with emotionally negative aggressiveness^1,12^. Consistent with the previous findings on the effect of sexual dimorphism on perceived attractiveness, Experiment 1 confirmed that attractiveness for computer-generated East-Asian faces negatively correlated with perceived masculinity for both male and female faces, indicating the preference for facial femininity in attractiveness judgments.

Despite the correlational nature of attractiveness and sexual dimorphism, Experiment 2 revealed that data-driven mathematical modelling and vector orthogonalisation technique allowed for independent manipulation of facial attractiveness and sexual dimorphism for both male and female faces. The validation test demonstrated that for the faces transformed along the attractiveness orthogonal to sexual dimorphism dimension, perceived attractiveness was changed as a function of the model-based face exaggeration while perceived masculinity/femininity remained unchanged. In contrast, for the faces transformed along the sexual dimorphism orthogonal to attractiveness dimension, perceived masculinity/femininity was changed as a function of the model-based face exaggeration while perceived attractiveness remained unchanged. These results, to our best knowledge, are the first to demonstrate the independent manipulation of perceived attractiveness and sexual dimorphism for male and female faces.

Of particular importance is that neither the effect of facial averageness nor symmetry explained the attractiveness dimension controled for sexual dimorphism. Indeed, facial averageness was U-shaped relationships with face exaggeration along the attractiveness orthogonal to sexual dimorphism dimension (Figure S1), suggesting that the face exaggeration made a face attractive and away from average faces at the same time. In addition, all the faces were symmetric; therefore facial symmetry was unavailable as a cue to attractiveness in this study. Taken together, the attractiveness dimension we exhibited here was not reduced to any effect of averageness, symmetry, and sexual dimorphism, which have been so far identified as the important cues to attractiveness^3,6^.

The hypothesis-driven studies on facial attractiveness have so far shown that facial attractiveness is judged through a combination of multiple facial cues, such as averageness, symmetry, and sexual dimorphism^4,5^. As such, the hypothesis-driven studies enable researchers to test the effect of a specific and predetermined feature on attractiveness, but such an approach tends to overlook other potentially important cues that the researchers have not taken into account^26^. Meanwhile, the data-driven approach has a great potential to discover as yet unidentified cues to attractiveness. As demonstrated by Said and Todorov^14^, perceived attractiveness is predictable with more precision by data-driven computational modelling than by a combination of averageness and sexual dimorphism. In our study, visual inspection of the facial features indicated that several facial features are related to perceived attractiveness and sexual dimorphism, respectively. For example, vertically wider eyes, a longer nose, and a sharper face contour appear to be characteristic of relatively attractive faces, while upturned eyes and eyebrows, a slight beard, and a darker skin appear to be associated with masculinity.

The independent manipulation of attractiveness and sexual dimorphism has important implications for the perception of facial attractiveness. First, even after the facial cues to sexual dimorphism are held constant, there remained critical cues to perceived attractiveness. Consistent with Said and Todorov^14^, facial variations we identified (Fig. 4) might reflect the as yet unidentified features, although we did not identify what exactly the features were. Of importance, the attractiveness cues after controlling for sexual dimorphism has a larger impact on the attractiveness judgements of male faces than female faces^28^. This indicates that the perceived attractiveness of female faces is determined by the sexual dimorphism to a larger degree, and that of male faces is rather evaluated the other facial cues than sexual dimorphism. Although it is beyond the scope of this study to identify what makes a face attractive after controlling for sexual dimorphism, several possible factors might be reflected in the face variations on the attractiveness orthogonal to sexual dimorphism dimension. One possible factor can be the familiarity to facial features. Faces similar to those previously seen are often judged as familiar and attractive^34–36^. Given the familiarity effect on perceived attractiveness, it is possible that the facial features varying on the attractiveness dimension reflect facial preferences of Japanese observers formed through prior exposure to typical facial features of their population^37^. Another possible factor is the desired personality inferred from faces^21,38^. People make trait inferences from facial appearance, and desirable personalities for a potential romantic partner or peers have a positive effect on perceived attractiveness^21,38^. Although sexual dimorphic facial features are cues to trait inferences^21^, the facial features unrelated to sexual dimorphism also affects trait impressions (e.g., extroversion and agreeableness^29^). It is possible that the face variation along the attractiveness orthogonal to sexual dimorphism dimension might be derived from trait inferences from facial appearance. Further empirical research awaits for clarifying what makes a face attractive after controlling for sexual dimorphism and what are the biological underpinnings.

Second, our findings can reconcile some aspects of controversial evidence on the effect of sexual dimorphism on perceived attractiveness^12,13,16,18–20,39^. As mentioned in the Background section, the previous studies often found facial dimorphism affected perceived attractiveness, but the effects varied across participants and methodologies. It is well-reported that the preference for the sexually dimorphic features depends on the perceiver’s characteristics such as the menstrual cycle^40^, perceiver’s own facial attractiveness^41^, sexual strategies^42,43^, and cultures^44,45^. More fundamentally, however, some of such variable results can be partially explained by the biased selection of facial images. That is, face manipulation of sexual dimorphism may alter the other aspect of facial cues affecting perceived attractiveness^20^. As our results revealed, the attractiveness cues orthogonal to sexual dimorphism have a profound impact on perceived attractiveness. It is thus possible that the effect of sexual dimorphism on perceived attractiveness can be confounded with that of the other factors orthogonal to sexual dimorphism. Indeed, the facial features unrelated to sexual dimorphism have impacts on various trait impressions such as extroversion and agreeableness^29^. In contrast, it is also possible that studies would confuse the effect of sexual dimorphism with that of attractiveness^46^. Future studies can investigate the true preference of sexual dimorphism or the genuine effects of attractiveness by using well-controlled facial stimuli like those we generated.

Despite our novel evidence that perceived attractiveness and sexual dimorphism are dissociable, there are several issues to be considered here. First, we have to keep in mind that even though the data-driven independent manipulation is possible, it does not necessarily mean that the faces generated from our data-driven models reflect the natural variations in the real world. It is natural that moderate or high correlation between facial attractiveness and femininity is observed when sampling a variety of real faces, as observed in previous studies^1,2^. Nevertheless, it is of importance that perceived attractiveness and sexual dimorphism, which highly correlate in natural face variations, can be changeable independently. Another limitation is that we used computer-generated faces for precise standardisation of facial features and experimental control over the multivariate features. However, computer-generated faces may lack some variations present in real-life human faces, such as skin smoothness, coloration, and blemishes^47^. Our findings should be validated in future studies using realistic photographs of human faces.

In conclusion, our data-driven mathematical model enabled generating faces whose perceived attractiveness and sexual dimorphism are independently manipulated. We were thereby able to leave out facial features that make a face attractive and masculine/feminine, respectively. Our findings will be of benefit to the further understanding of what makes a face attractive and why people hold a preference for such facial features.

## Supplementary information

Supplementary information.

## Data Availability

Raw data, an R code for the main analysis, and visual illustration of the facial transformation presented in the current study are available from the Dryad Digital Repository (10.5061/dryad.pg4f4qrmp).
