# Supplementary material for: A new data-driven mathematical model dissociates attractiveness from sexual dimorphism of human faces
Source: Sci Rep. 2020 Oct 6;10:16588. doi: 10.1038/s41598-020-73472-8 (PMC7538911; doi:10.1038/s41598-020-73472-8)
Supplement: Supplementary file 1 — Supplementary information. [file 41598_2020_73472_MOESM1_ESM.docx]

**A new data-driven mathematical model dissociates attractiveness from sexual dimorphism of human faces**

Koyo Nakamura^1,2,3*^, and Katsumi Watanabe^1,4^

^1^Faculty of Science and Engineering, Waseda University, Tokyo

^2^Japan Society for the Promotion of Science, Tokyo

^3^Keio Advanced Research Centers, Tokyo

^4^Art & Design, University of New South Wales, Sydney

*Correspondence concerning this paper should be addressed to Koyo Nakamura, Faculty of Science and Engineering, Waseda University, 3-4-1, Ohkubo, Shinjuku, Tokyo, 169-8555, Japan.

Email: [koyo@fennel.sci.waseda.ac.jp](mailto:koyo@fennel.sci.waseda.ac.jp)

Table S1. The Bayesian hierarchical models and the WAIC

| Rating | Face transformation | Fixed effects | WAIC (Models without the face exaggeration quadratic terms) | WAIC  (Models with the face exaggeration quadratic terms) |
| --- | --- | --- | --- | --- |
| Sexual dimorphism (Experiment 1) | Sexual dimorphism | Face exaggeration | 7094.80 | 6993.78 |
|  |  | Face exaggeration X Sex of raters | 7094.90 | 6994.37 |
|  |  | **Face exaggeration X Sex of faces** | 6968.08 | 6862.01 |
|  |  | Face exaggeration X Sex of faces X Sex of raters | 6969.52 | 6864.94 |
| Attractiveness  (Experiment 2) | Attractiveness orthogonal to sexual dimorphism | Face exaggeration | 7299.90 | 7229.44 |
|  |  | Face exaggeration X Sex of raters | 7299.94 | 7230.85 |
|  |  | **Face exaggeration X Sex of faces** | 7174.12 | 7101.69 |
|  |  | Face exaggeration X Sex of faces X Sex of raters | 7174.51 | 7105.68 |
| Sexual dimorphism  (Experiment 2) | Attractiveness orthogonal to sexual dimorphism | Face exaggeration | 8112.94 | 8110.23 |
|  |  | Face exaggeration X Sex of raters | 8113.77 | 8112.14 |
|  |  | **Face exaggeration X Sex of faces** | 7919.86 | 7919.60 |
|  |  | Face exaggeration X Sex of faces X Sex of raters | 7920.37 | 7920.60 |
| Attractiveness  (Experiment 2) | Sexual dimorphism orthogonal to attractiveness | Face exaggeration | 8494.62 | 8492.96 |
|  |  | Face exaggeration X Sex of raters | 8495.11 | 8493.88 |
|  |  | **Face exaggeration X Sex of faces** | 8402.54 | 8402.02 |
|  |  | Face exaggeration X Sex of faces X Sex of raters | 8402.86 | 8405.92 |
| Sexual dimorphism  (Experiment 2) | Sexual dimorphism orthogonal to attractiveness | Face exaggeration | 7733.77 | 7733.77 |
|  |  | Face exaggeration X Sex of raters | 7733.04 | 7690.38 |
|  |  | **Face exaggeration X Sex of faces** | 7442.59 | 7391.79 |
|  |  | Face exaggeration X Sex of faces X Sex of raters | 7442.37 | 7393.27 |

Note. The bolded models were selected as the best-fitted models in this study.

Figure S1. Facial averageness of the male and female faces exaggerated along (i) the attractiveness orthogonal to sexual dimorphism dimension (indicated in red) and (ii) the sexual dimorphism orthogonal to attractiveness dimension (indicated in blue). Colored dots and error bars indicate the mean Euclidean distances from the average face and the standard errors. Facial averageness is quantified by calculating the Euclidean distance of 100 facial feature dimensions (50 shape and 50 reflectance dimensions) of the individual faces and the average faces across the 200 original faces which were used in Experiment 1, separately for male and female faces.
